# Gene Loss Predictably Drives Evolutionary Adaptation

**DOI:** 10.1093/molbev/msaa172

**Published:** 2020-07-13

**Authors:** Jana Helsen, Karin Voordeckers, Laura Vanderwaeren, Toon Santermans, Maria Tsontaki, Kevin J Verstrepen, Rob Jelier

**Affiliations:** m1 Laboratory of Predictive Genetics and Multicellular Systems, CMPG, KU Leuven, Leuven, Belgium; m2 Laboratory of Genetics and Genomics, CMPG, KU Leuven, Leuven, Belgium; m3 Laboratory for Systems Biology, VIB-KU Leuven Center for Microbiology, Leuven, Belgium

**Keywords:** genetic network, adaptation, experimental evolution, fitness landscape, oxidative stress, evolvability

## Abstract

Loss of gene function is common throughout evolution, even though it often leads to reduced fitness. In this study, we systematically evaluated how an organism adapts after deleting genes that are important for growth under oxidative stress. By evolving, sequencing, and phenotyping over 200 yeast lineages, we found that gene loss can enhance an organism’s capacity to evolve and adapt. Although gene loss often led to an immediate decrease in fitness, many mutants rapidly acquired suppressor mutations that restored fitness. Depending on the strain’s genotype, some ultimately even attained higher fitness levels than similarly adapted wild-type cells. Further, cells with deletions in different modules of the genetic network followed distinct and predictable mutational trajectories. Finally, losing highly connected genes increased evolvability by facilitating the emergence of a more diverse array of phenotypes after adaptation. Together, our findings show that loss of specific parts of a genetic network can facilitate adaptation by opening alternative evolutionary paths.

## Introduction

Loss of gene function is common in nature. An average human, for example, carries about 100 loss-of-function variants in their genome (MacArthur et al. 2012). Even though loss-of-function mutations can lead to genetic disorders and reduced fitness (Stenson et al. 2017), they have also been proposed to be an important source of phenotypic diversity in evolution (Olson 1999; Meredith et al. 2014; Albalat and Cañestro 2016; Peter et al. 2018; Sharma et al. 2018). When a budding yeast cell loses a gene, the effect on fitness in any given condition can vary from beneficial to lethal (Giaever et al. 2002). This pattern of essentiality and dispensability also depends on the genetic background, with some genes being essential in one genotype and dispensable in another (Dowell et al. 2010; Liu et al. 2015). Similarly, the phenotypic effect of deleting a gene can depend on which other genes have been inactivated, a phenomenon that has been used extensively to systematically map interactions between genes and construct the genome-scale genetic interaction network of budding yeast (Costanzo et al. 2010). Given that the effect of one mutation can depend on the presence or absence of other mutations also implies that in some cases, the negative fitness effects resulting from gene loss can be mitigated by other compensating mutations. Over the years, it has been shown that the way in which cells adapt to gene loss can be linked to the functional effect of the lost gene (Rancati et al. 2008; Szamecz et al. 2014; Laan et al. 2015; McCloskey et al. 2018; Rojas Echenique et al. 2019). For example, budding yeast cells lacking *BEM1*, a gene essential for cell polarity, restore wild-type polarization by modulating the activity of its interaction partner Cdc42 (Laan et al. 2015). However, reports that link the function of the lost genes with the mutations acquired during adaptation are based either on small, case-specific experiments (Rancati et al. 2008; Laan et al. 2015) or on larger-scale screens where convergent evolution between strains with defects in similar processes was rare, which precludes drawing strong general conclusions on the adaptive routes and overall evolutionary consequences of gene loss (Szamecz et al. 2014; Rojas Echenique et al. 2019). In addition, genes are not stand-alone units but rather function in a coordinated manner in genetic networks. Including information about an organism’s genetic network architecture is crucial for understanding how and why a cell compensates in a specific way for the loss of a particular gene.

Here, we systematically explored the influence of genetic architecture on adaptation after gene loss and evaluated how gene loss affects the speed of adaptation and evolvability, that is, the ability of the organism to produce heritable—potentially adaptive—phenotypic variation (Hansen 2006). We focused on genes important for resistance to oxidative stress, a trait involved in multiple disease phenotypes, including cancer, neurodegenerative disorders, and age-related diseases (Barnham et al. 2004; Reuter et al. 2010). After identifying specific genes important for oxidative stress resistance in Saccharomyces *cerevisiae*, we evolved more than 200 strains in which one of these key genes was deleted under oxidative stress and investigated whether and how they were able to overcome their respective defects. This experimental setup allowed us to explore whether strains that lack genes from different modules in the genetic network adapt through distinct evolutionary routes and if by doing so, they end up in a different place in the fitness landscape.

## Results

### Characterizing Genetic Network Architecture

**Fig. 1.**
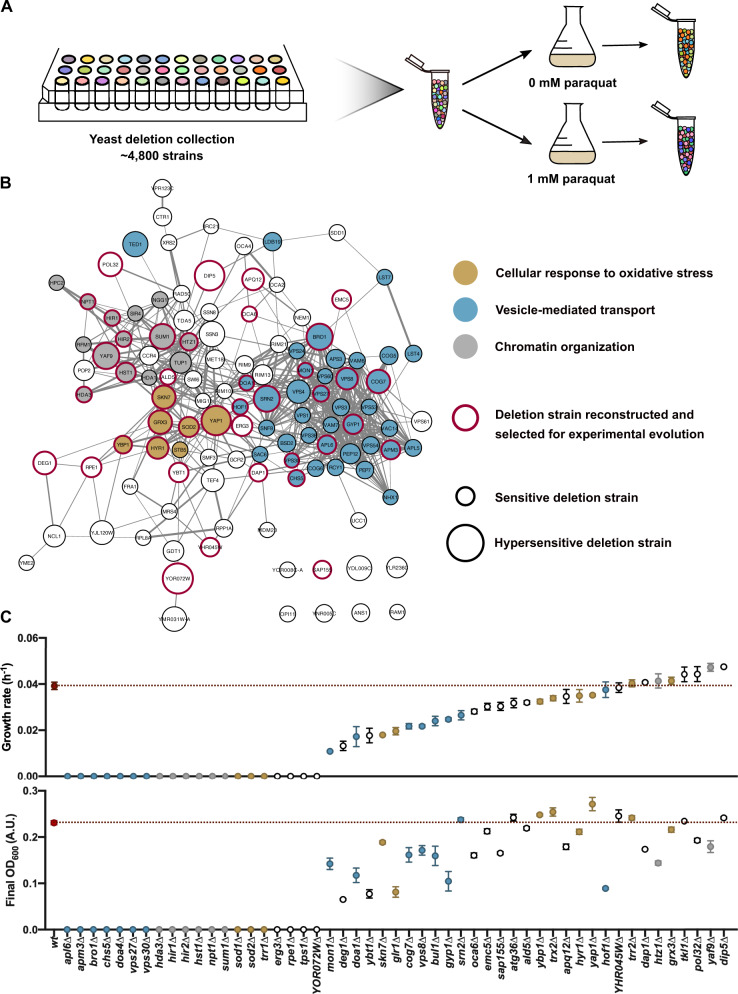
A comprehensive genetic screen identifies cellular processes important for resistance to paraquat stress. (*A*) Schematic overview of the experiment. All the deletion strains from the haploid deletion collection were pooled, and the pool was grown with and without paraquat to determine the relative fitness of each individual mutant under paraquat stress. (*B*) Interaction network with the genes that, when deleted, increase sensitivity to paraquat (log FC < −3.5). Three of the most enriched cellular processes are highlighted in gray (chromatin organization), gold (cellular response to oxidative stress), and blue (vesicle-mediated transport). The size of the nodes represents the sensitivity to paraquat as determined in the genome-wide screen. The thickness of the edges represents the confidence score associated with the interaction as determined by STRING. Nodes with red borders represent genes for which deletion mutants were made and which were selected for experimental evolution. (*C*) Growth rates and final OD_600_ values after 120 h of deletion strains selected as starting strains for experimental evolution on YP 2% (w/v) glycerol with 0.125 mM paraquat. Error bars represent SEM of four replicate measurements. Strains are colored based on the cellular process they belong to: gray for chromatin organization, gold for cellular response to oxidative stress, and blue for vesicle-mediated transport.

To systematically assess which genes are important for growth under oxidative stress, we determined the relative fitness of each mutant in the haploid yeast deletion collection (Giaever et al. 2002) in the presence of paraquat, a commonly used superoxide generator (Fukushima et al. 2002). As each deletion strain contains unique DNA barcodes, strains can be pooled and their relative frequencies can be determined by isolating and sequencing the barcodes (Smith et al. 2009). We grew a pool containing all ∼4,800 deletion strains from the haploid deletion collection on rich medium with and without paraquat (fig. 1*A*). Next, we calculated the fitness of each strain based on its relative number of barcodes in each condition. Strains with the lowest fitness under paraquat stress lacked genes involved in three main cellular processes: cellular response to oxidative stress, vesicle-mediated transport, and chromatin organization (fig. 1*B* and supplementary tables 1 and 2, Supplementary Material online). Conversely, the large majority of strains with a higher fitness under paraquat stress missed genes involved in mitochondrial function (supplementary fig. 1 and supplementary tables 1 and 2, Supplementary Material online). This is probably due to the fact that paraquat primarily generates superoxide radicals in the mitochondrial matrix (Cochemé and Murphy 2008). Because yeast cells are able to survive with absent or defective mitochondria (Ephrussi et al. 1949), loss of mitochondrial function would be a simple and predictable mechanism of adaptation. To prevent this, we forced the cells to adapt while respiring by using glycerol as a carbon source instead of glucose (Gancedo et al. 1968). We then selected 48 genes whose deletions caused a large fitness decrease in the genome-wide screen (fig. 1*B*) together with three genes that in literature have been shown to be central players in oxidative stress resistance (supplementary table 3, Supplementary Material online). Finally, we measured the growth characteristics of each of these strains on rich medium with glycerol and paraquat, ending up with 51 different deletion strains showing varying degrees of sensitivity: from mutants that did not show any observable growth within the sampling window, to mutants whose growth characteristics were similar to those of the wild type (fig. 1*C*). Growth rates were used as a proxy for fitness (Szamecz et al. 2014; McCloskey et al. 2018). We decided to include each of these 51 mutants for experimental evolution, even those that did not show decreased fitness under paraquat stress. This way, we are also able to evaluate the effect of gene loss on evolution even without obvious fitness effects.

### Gene Loss Can Speed up Evolution

**Fig. 2.**
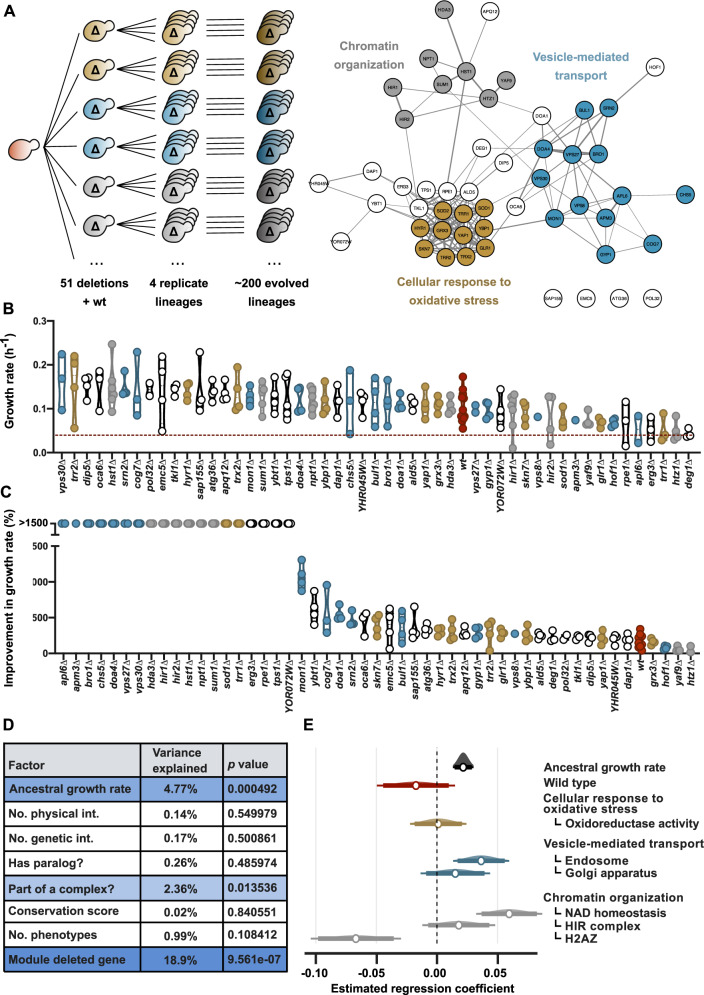
Deletion strains greatly improve growth during adaptive evolution. (*A*) Schematic overview of the evolution experiment and gene network with all selected genes. Strains with deletions in different modules of the genetic network underlying resistance to oxidative stress are evolved in quadruplicate under paraquat stress for 150 generations. Genes in the network are colored corresponding to the indicated cellular processes they belong to. The color scheme is maintained throughout the figure. (*B*) Growth rates of one fit clone from each evolved population on rich medium with 2% (w/v) glycerol and 0.125 mM paraquat. Each point represents the mean of four replicate measurements. The deletion strains are sorted according to their average growth rate after evolution. The red dotted line represents the average growth rate of the wild type before evolution. (*C*) Improvement in growth rate of one fit clone for each evolved population on rich medium with glycerol and paraquat. (*D*) Result of a multivariate linear model analysis of the growth rate after evolution in function of various biological parameters. Shades of blue correspond with significance level. (*E*) Estimated regression coefficient per network module of the deleted gene with their 95% confidence intervals (multivariate linear regression).

We then explored how strains with defects in different cellular processes adapt to chronic oxidative stress and whether this ability depends on which gene was lost initially. To do this, we evolved each of the 51 deletion strains in quadruplicate for ∼150 generations (fig. 2*A*). In addition, we evolved 12 replicates of the wild type, amounting to a total of more than 200 independent lineages. First, as a control, we evolved the strains in standard glucose medium with paraquat. As expected, the vast majority of strains showed a significant loss of mitochondrial function after 150 generations, confirming that the paraquat treatment causes oxidative stress that mainly targets the mitochondria (supplementary fig. 2*A*, Supplementary Material online). Next, we repeated the experiment in medium with paraquat and glycerol as the sole carbon source, thereby selecting against loss of mitochondrial function because glycerol metabolism depends on respiration. At the end of the evolution experiment, the majority (80%) of the lineages showed growth (supplementary table 4, Supplementary Material online). Only for one deletion strain (*sod2*Δ) did none of the replicates produce viable cells at the end of the experiment. This indicates that *SOD2* is indeed an essential gene for survival under oxidative stress in conditions that select against loss of mitochondrial function. Next, we selected one fit clone from each of the 202 surviving populations (190 deletion strains and 12 wild-type strains) and measured its growth on the selective medium. The vast majority of the evolved lineages showed a dramatic increase in fitness compared with the respective unevolved deletion mutants (fig. 2*B* and *C* and supplementary fig. 2*B* and *C*, Supplementary Material online). Several evolved deletion strains (13 out of 190) reached a higher fitness than the most rapidly adapting wild-type strain. Surprisingly, when comparing the average absolute growth rates between strains, a large fraction of evolved deletion strains performed better than the average evolved wild type, even though some of these strains initially showed severe growth defects (fig. 2*B* and *C*). We tested whether there are indeed strains that perform significantly better than the wild type, versus the null hypothesis that the higher than average wild-type fitness values can be explained by assuming the wild-type fitness distribution for all strains. The test showed that this null hypothesis can be rejected (*P* < 0.001; supplementary fig. 2*D*, Supplementary Material online), and we conclude that the expected growth rates of several strains are indeed higher than wild type. We further find that the result is robust and does not rely on a few outliers (supplementary fig. 2*D*, Supplementary Material online). Finally the result does not depend on lower initial fitness of the deletion strains, as it also holds for the subset of deletion strains with growth rates that are initially similar to the wild type (supplementary fig. 2*E*, Supplementary Material online).

Next, we explored which biological parameters could explain the observed growth rate after evolution. In agreement with previous studies (Wiser et al. 2013; Rojas Echenique et al. 2019), we found a significant positive relationship between the original growth rate and the growth rate after evolution (fig. 2*D*). However, the location of the originally deleted gene in the genetic network proved to be a much better predictive factor of a mutant’s ability to adapt to oxidative stress (fig. 2*D*). Strains with deletions in particular submodules, such as NAD homeostasis or the H2AZ histone variant within the chromatin organization module, respectively, performed significantly better and worse than the average evolved strain, irrespective of the growth rate of their ancestors (fig. 2*E*). Importantly, strains with a deletion in the NAD homeostasis submodule or the endosome submodule also reached higher mean fitness levels than evolved wild-type strains (*P* < 0.05, Mann–Whitney nonparametric tests). Together, these results show that gene loss can speed up evolution in a genotype-dependent way, even to levels higher than the average evolved wild type.

### Mutational Patterns Reflect Genetic Network Architecture

**Fig. 3.**
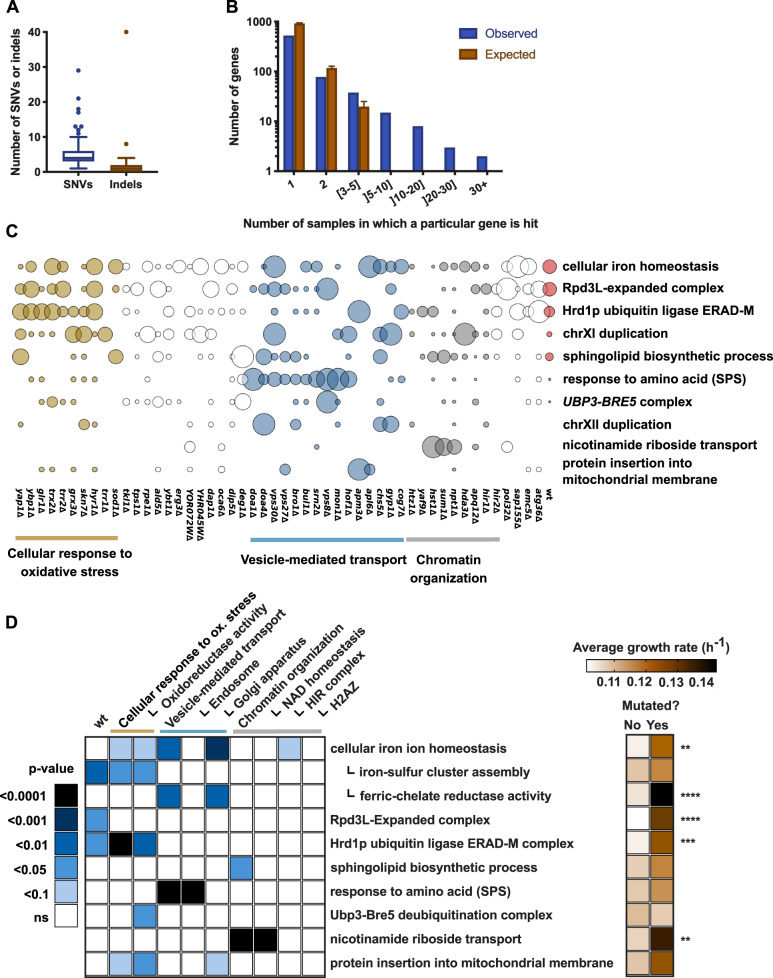
Evolution is convergent at the level of modules and individual genes. (*A*) Number of SNVs and indels per sequenced sample. (*B*) Number of times a particular gene is hit across the sequenced samples, compared with what would be expected by chance. (*C*) Overview of the cellular processes (GO terms) that are mutated more often than expected by chance across all samples (supplementary table 7, Supplementary Material online), and two of the most frequently duplicated chromosomes. The size of the circles represents the fraction of independently evolved clones with a mutation in the indicated gene or pathway. (*D*) Specificity of each mutated process for the original genetic background and its influence on growth rate. The left side of the figure shows how often each mutated process (rows) is found within the original genetic backgrounds (columns), the latter of which are represented by genetic modules of the deleted genes. *P*-values represent enrichment scores and were calculated using a competitive gene set overrepresentation test as calculated by the camera function of the edgeR package. The right side of the figure shows the effect of having a mutation in one of these processes on the growth rate of the evolved strains. The average growth rates are calculated across all evolved lineages. **P < 0.01, ***P < 0.001, ****P < 0.0001 (Student’s *t*-test).

We then went on to investigate whether gene loss in distinct cellular processes causes strains to compensate by acquiring mutations in distinct pathways. We sequenced the previously selected fit evolved clones (190 deletion strains and 8 wild-type strains), together with the ancestor wild type, and identified the mutations that were acquired during adaptation. On average, strains contained five single nucleotide variants (SNVs) and one indel, the majority of which were, respectively, nonsense mutations or mutations that cause a frameshift (fig. 3*A*, supplementary fig. 3 and supplementary table 5, Supplementary Material online). About half of the evolved strains (51%) had aneuploidies, and 9% increased total ploidy (supplementary table 6, Supplementary Material online). Across samples, we observed a remarkably large number of genes that were hit repeatedly (fig. 3*B*). This strong signature of parallel evolution became even clearer when looking at the level of cellular processes. By doing a Gene Ontology (GO) enrichment test across all samples, we found several distinct processes that are mutated much more often than expected by chance and thus are very likely to have been subject to positive selection (fig. 3*C* and supplementary table 7, Supplementary Material online). Next, we examined whether the mutations can be linked to the process that was perturbed by the initial deletion. By performing targeted GO enrichment tests per genetic module, we indeed found strong patterns of such module-specific mutation patterns (fig. 3*D* and supplementary table 8, Supplementary Material online). For example, mutations involved in nicotinamide riboside transport were almost exclusively found in strains with defects in NAD homeostasis (*P* < 0.0001) and mutations involved in the response to amino acids (SPS) were specific to strains with defects in endosomal vesicular transport (*P* < 0.0001) (fig. 3*C* and *D*). There were also some examples where adaptive mutations could be linked to a single gene instead of to a module. For example, every *sod1*Δ strain acquired mutations in genes involved in Mn^2+^ transport (supplementary table 5, Supplementary Material online), an adaptive mechanism which has been previously reported (Elashvilis et al. 1992). In addition to mutations being specific to the module of the deleted gene, some of them also had a larger effect on the growth rate of the evolved strain than others (fig. 3*D*). Some genotypes were more prone to acquiring mutations with such a large effect, which could be part of the explanation of why some strains in the end do better than the wild type.

**Fig. 4.**
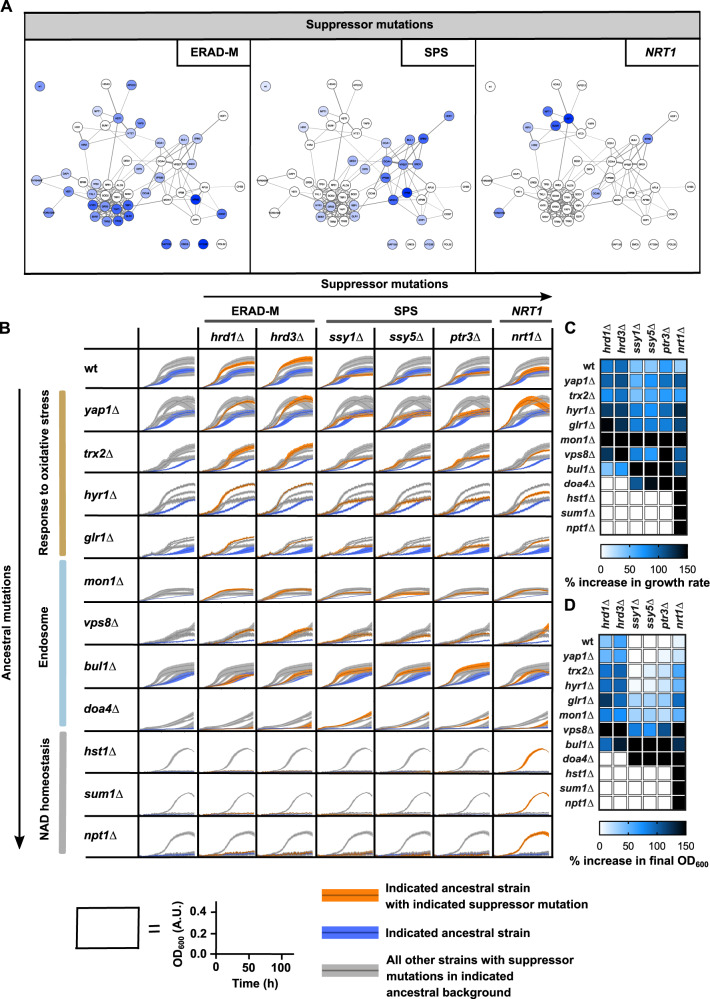
Validation of module-specific adaptation. (*A*) Mutations in ERAD-M, SPS, or *NRT1*, projected onto the original gene deletion network with genes important for resistance to paraquat stress. The intensity of the colors corresponds to the relative frequency of individually evolved clones with a mutation in the indicated complex, pathway or gene. (*B*) Growth curves of double deletion mutants (ancestral deletion and adaptive deletion) on YP 2% (w/v) glycerol with 0.125 mM paraquat. Each row contains one of the original genetic backgrounds that were used during experimental evolution, and each column contains deletions in genes that were indicated to be adaptive. Dark blue curves represent the growth of the ancestor strain without any other additional deletions (first column), orange curves correspond to the growth of the double deletion strain indicated by the row and column headings, and gray curves represent growth curves of all other strains within the same row. Error bars represent the standard deviation of four replicates. (*C*) Heat map with the increase in growth rate for each combination of deletions. (*D*) Heat map with the increase in final OD_600_ for each combination of deletions.

To further investigate and validate whether the compensatory mutations that were acquired during experimental evolution are indeed linked to loss-of-function mutants in particular cellular functions, we chose three processes with the strongest specific signature (ERAD-M: endoplasmic-reticulum [ER]-associated protein degradation; SPS, amino acid sensing; *NRT1*: transporter for nicotinamide riboside) (fig. 4*A*) and deleted the mutated genes in the ancestral (unevolved) deletion strains. The mutations that were observed in these genes were predicted to cause loss of function, so we reasoned that a gene deletion would have a similar effect. Each of these gene deletions did indeed increase fitness in their corresponding deletion background (fig. 4*B*–*D*). For example, suppressor mutations in *NRT1* were primarily found in *hst1*Δ, *sum1*Δ, and *npt1*Δ, three strains that are involved in NAD homeostasis. In these ancestral backgrounds, deleting *NRT1* was indeed the only tested mutation that managed to improve growth. Interestingly, each of the tested deletions also showed a positive growth effect in genetic backgrounds in which this mutation was not observed during evolution. For example, mutations in SPS or *NRT1* increased fitness in the *trx2*Δ background, yet this combination of mutations never occurred during evolution. In general, suppressor mutations with the largest beneficial effect on growth seem to have been favored by selection (fig. 4*B*–*D*).

### Losing Hub Genes Increases Evolutionary Variability

**Fig. 5.**
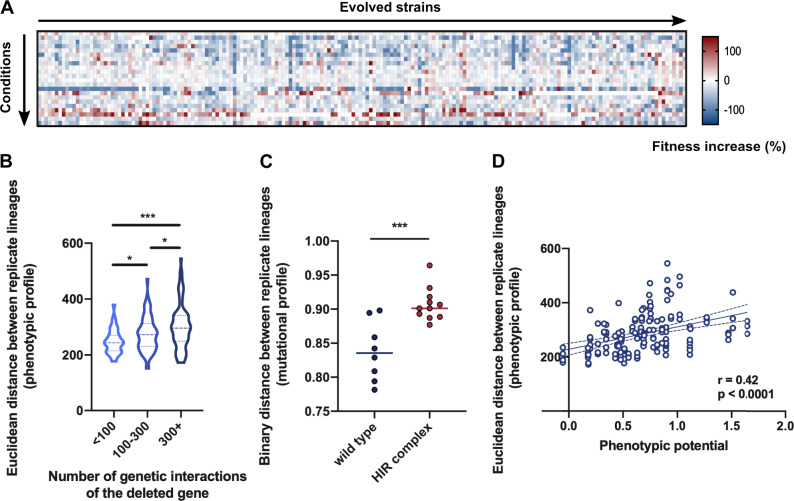
Adaptation after losing genes with more interaction partners results in a higher phenotypic variability between independent lineages. (*A*) Heat map with the fitness increase (%) of the evolved strains (columns) across 22 different phenotypic conditions (rows). Red hues indicate a fitness increase as compared with the corresponding strain before evolution and blue hues indicate a decrease in fitness. (*B*) Phenotypic distance between replicate lineages in function of the number of genetic interactions of the deleted gene. A high value for the phenotypic distance corresponds with more variation between the phenotypic profiles of the replicate lineages of a particular deletion strain. *P < 0.05, ***P < 0.001 (Student’s *t*-test). (*C*) Mutational distance between replicate lineages of the wild-type strains compared with strains lacking genes from the HIR complex. The mutational profile is defined by the genes and GO categories that were mutated after evolution. A high value for the mutational distance corresponds with more variation between the mutational profiles of the replicate lineages of a particular deletion strain. ***P < 0.001 (Student’s *t*-test). (*D*) Phenotypic distance between replicate lineages in function of phenotypic potential, as determined in Levy and Siegal (2008). The phenotypic potential represents the overall phenotypic variation when a gene is deleted. Lines represent the best linear fit with its 95% confidence interval.

Apart from its modular structure, another important feature of the architecture of genetic networks is the number of physical and genetic interactions of each gene. It has been hypothesized that losing genes with many connections (so-called hubs) would lead to a more diverse evolutionary outcome (Koubkova-Yu et al. 2018; Helsen et al. 2019). We therefore determined the phenotypic profile of the evolved strains across 22 different environments (fig. 5*A* and supplementary table 9, Supplementary Material online). In order to quantify the phenotypic variation between strains, we determined the average distance between phenotypic profiles for each strain carrying the same gene deletion. This measure represents the diversity of the evolutionary outcome for each genotype. Interestingly, we found that the phenotypic distance between replicate lineages varied with the number of genetic interaction partners of the original gene deletion. On average, lineages that evolved from a mutant in which a gene with more genetic interactions (i.e., a hub) was deleted showed more variability in their phenotypic profiles (fig. 5*B*). To investigate whether this increase in phenotypic variability was linked to an increased mutational variability, we determined the variation in the mutational spectrum of the evolved lineages that lacked the same gene. Here too, variability increased with the number of genetic interactions of the originally deleted gene, but only marginally (supplementary fig. 4, Supplementary Material online). Instead, the increase we do observe for genes with a lot of genetic interactions is the result of a specific subset of hub gene deletion mutants, such as strains with deletions in the HIR complex (fig. 5*C*). Strains lacking genes with many interaction partners have previously also been shown to exhibit a higher level of within-strain phenotypic variation (Levy and Siegal 2008). In other words, when such genes are deleted, a population grown from these deletion strains will show more phenotypic variability between individual cells. Remarkably, this measure, also known as phenotypic potential, showed a significant positive correlation with the phenotypic distance between replicate lineages (fig. 5*D*). This could imply that a strain with a gene deletion that generates more variability between individuals can evolve in various ways and give rise to descendants with a wide range of phenotypes across different environments.

## Discussion

Our results show how gene loss can be compensated and can ultimately even facilitate and enhance adaptation. Furthermore, the results also reveal the importance of genotype and genetic architecture during adaption to gene loss. Specifically, the evolutionary fate of a deletion strain depends on the molecular function of the deleted gene and its place in the genetic network, as this strongly influences the direction and variation of the mutational trajectories during evolution. For example, strains lacking genes involved in NAD homeostasis acquired mutations in *NRT1*, a transporter of the NAD precursor nicotinamide riboside. Deleting *NRT1* also has a positive—albeit smaller—effect on growth in other genetic backgrounds, which indicates that NAD balance plays a general role in protecting cells against paraquat stress. On the other hand, mutations in ERAD-M were primarily found in strains that lost genes important for the primary response to oxidative stress. Paraquat has previously been shown to attenuate the unfolded protein response (UPR) in the ER, and this effect is even stronger specifically in strains that have lost genes involved in the response to oxidative stress (Maity et al. 2016). The ERAD-M machinery is known to degrade *IRE1* (Sun et al. 2015), the sole sensor and activator of UPR in the ER in yeast (Shamu and Walter 1996), so inactivating ERAD-M might help restore the UPR.

Apart from yielding insight into the evolutionary role of gene loss, comparing the mutational profiles between evolved strains also allows us to gain insight into the organization of the genetic network. For example, among the genes involved in vesicular transport, we can easily distinguish which genes are endosomal and which ones belong to the Golgi apparatus, by determining whether their evolved deletion strains carry an adaptive mutation in SPS. Additionally, exploring which processes are able to compensate for loss of function in other processes provides a novel insight into the larger-scale hierarchy of cellular processes. Although additional experiments are necessary to unravel why certain cellular processes can compensate for loss of function in a particular module of the genetic network, it is clear that both NAD balance, UPR and amino acid homeostasis play an important role in the response to paraquat stress.

Our findings further show that losing a gene can increase both an organism’s speed of adaptation and evolvability, by increasing the variability of phenotypes that emerge during adaptation. Perhaps surprisingly, the loss of well-connected genes that take a central position in a genetic network tends to generate lineages during evolution with a high diversity in phenotypic profiles. The loss-of-function of such hub genes could therefore improve the evolutionary potential in situations that demand rapid and multifaceted innovation. In complex environments where cells are forced to quickly adapt multiple phenotypes, the loss of hub genes leads to phenotypic variability, which in turn may facilitate further adaptation. Interestingly, the evolutionary potential and speed of adaptation also depend on the deleted gene, with deletions in some functional modules generally leading to a quicker and stronger recovery, sometimes even to levels higher than the evolved wild-type strains.

Why do cells that lack a gene sometimes evolve faster and to higher fitness values than strains that did not lose a gene? It is clear that a gene deletion can change the evolutionary path of a strain. A lower fitness due to a gene deletion may make a further mutation more likely to be adaptive. To describe the situation in an intuitive way, imagine a fitness landscape with different peaks and valleys (Wright 1932). In the case of a relatively fit wild-type strain, individuals may be trapped near a local fitness peak with selective pressure keeping them near the peak. Losing a gene may cause dramatic changes in both the landscape and the position of the individual in the landscape. If a gene deletion brings the strain to a fitness valley, individuals may then be able to reach other, higher peaks. Indeed, simulations with digital organisms have shown that deleterious mutations may serve as stepping stones in adaptive evolution (Covert et al. 2013).

It is important to note that in nature, the decrease in fitness associated with gene loss could impede the spread of the mutation in the population. Indeed, genes remain functional over long evolutionary periods for this reason. However, gene loss is pervasive across all forms of life, suggesting that at least in some circumstances, loss-of-function mutants survive (reviewed in Albalat and Cañestro 2016). Indeed, depending on the condition in which they occur, null mutations are often neutral and can even provide fitness benefits (Costanzo et al. 2010). Moreover, our results show that suppressor mutations that inactivate genes can help restore fitness levels quickly, confirming the results of previous directed evolution studies (Kvitek and Sherlock 2011; Hottes et al. 2013). Together with our finding that gene loss can increase the speed of adaptation and evolvability, it becomes clear how the loss of a gene can serve as a gateway to rapid adaptation and evolutionary innovation.

## Materials and Methods

### Strains

#### Construction of a Prototrophic S288c Strain with Repaired HAP1, SAL1, and MIP1

All strains used in the evolution experiments and growth assays are derived from the haploid prototrophic S288c strain FY4 (*MATa*) (Brachmann et al. 1998), in which *HAP1, SAL1*, and *MIP1* were repaired using CRISPR-Cas9. These three genes are important for faithful inheritance and functioning of mitochondria (Gaisne et al. 1999; Dimitrov et al. 2009; Ehrenreich et al. 2010) and are mutated in the standard S288c lab strain. Appropriate guide sequences were inserted in pV1382, a Cas9/sgRNA delivery plasmid which was a gift from the Gerald Fink Lab (Vyas et al. 2018). FY4 was then cotransformed with the plasmid and a repair template using the standard LiAc-based yeast transformation protocol, to sequentially repair *HAP1*, *SAL1*, and finally *MIP1*. Correct clones were identified using Sanger sequencing and later also confirmed using whole-genome sequencing. The new reference strain showed improved mitochondrial function and mitochondrial genome stability (supplementary fig. 5*A* and *B*, Supplementary Material online). A full list of strains used in this study can be found in supplementary table 10, Supplementary Material online.

#### Construction of Sensitive Deletion Strains

All deletion strains used in the evolution experiments were made in FY4 HAP1+ SAL1+ MIP1+ using the standard LiAc-based yeast transformation protocol. Genes were deleted using a loxP-HYG-loxP cassette based on a plasmid containing a HYG-resistance marker analogous to pUG6 (Christiaens et al. 2014). A full list of strains used in this study can be found in supplementary table 10, Supplementary Material online.

#### Construction of Validation Deletion Strains

All deletion strains used in the validation experiments were made in FY4 HAP1+ SAL1+ MIP1+ and a selection of sensitive deletion strains using the standard LiAc-based yeast transformation protocol. Deletion cassettes were based on pUG6, conferring resistance to G418 disulfate (Güldener et al. 1996). A full list of strains used in this study can be found in supplementary table 10, Supplementary Material online.

### Genome-Wide Screen

#### Pooling the Deletion Collection

The haploid MATa deletion collection was pooled as described in Perez-Samper et al. (2018). In short, the collection was thawed and 3 µl from each well was inoculated in 150 µl YP (20 g/l bacterial peptone, 10 g/l yeast extract) supplemented with 2% (w/v) glucose and 200 µg/ml G418. Cultures were grown to stationary phase (OD_600_ > 1.0) and 50 µl from each well was pooled and mixed. The pool was then distributed into 1-ml aliquots and frozen.

#### Growing the Pool and Sample Preparation

Two deletion collection pool aliquots were thawed and pregrown separately for one overnight in 50 ml YP 2% (w/v) glucose at 30 °C. A sample was procured for the initial time point, and for each replicate ∼11 x 10^4^ cells were transferred to 200 ml YP 2% (w/v) glucose and 200 ml YP 2% (w/v) glucose supplemented with 1 mM paraquat (Sigma-Aldrich). The pools were grown for ∼6 doublings at 30 °C, after which ∼11 x 10^4^ cells were transferred to fresh medium. This step was repeated twice, so that every pool had undergone ∼18 doublings while continuously growing in exponential phase before taking the final samples. Using a standard zymolyase-based protocol, genomic DNA was extracted from the two initial and four final samples. UPTAGs and DNTAGs were then amplified in separate PCR reactions using the primers described in Perez-Samper et al. (2018). UPTAG and DNTAG PCR mixtures coming from the same sample were pooled, and samples were sent for sequencing on an Illumina NextSeq 500.

#### Bioinformatic Analysis

Barcodes were extracted from the raw sequence reads using cutadapt version 1.12 (Martin 2011). Extracted barcodes were aligned to the reannotated deletion barcodes (Smith et al. 2009), using Barcas version 1.0 (Mun et al. 2016) and allowing for a maximum of two inexact matches (mismatch, deletion or insertion). Statistical tests to determine the differential abundance of each mutant were performed in edgeR version 3.8 (Robinson et al. 2010), using TMM normalization. The log_2_-fold change in barcode abundance (1 mM paraquat condition vs. 0 mM paraquat condition) for each gene deletion was used to rank the gene deletion strains based on their fitness. The camera function from edgeR was used to perform the GO enrichment analysis (Wu and Smyth 2012). Gene networks representing the most depleted and enriched deletion strains were made in STRING version 11.0 (Szklarczyk et al. 2015) and visualized using Cytoscape version 3.7.1 (Shannon et al. 2003).

### Growth Assays in Liquid Culture

All growth measurements reported in this study were done in liquid culture, except for those associated with the phenotypic screens (see below).

#### Growth Assays on YP 2% (w/v) Glycerol

For each tested strain, one colony was inoculated in triplicate in 150 µl YP 2% (w/v) glycerol + 1 µM CuSO_4_ and serially diluted for growth overnight at 30 °C and 900 rpm. Copper was added to improve respiratory growth (Schlecht et al. 2014). Cultures at OD_600_ < 0.1 were transferred to 150 µl of fresh medium and serially diluted for growth for another overnight. Finally, cultures at OD_600_ < 0.1 were selected and used to inoculate for growth measurements in a Bioscreen C device. The two consecutive precultures ensure that every culture is fully adapted and growing exponentially before the start of the growth experiment. Cells were incubated at 30 °C, with continuous medium shaking and OD_600_ was tracked every 15 min. Growth rates were determined using an in-house python script, and final OD_600_ measurements were obtained after 120 h of growth.

#### Growth Assays on YP 2% (w/v) Glycerol, Supplemented with 0.125 mM Paraquat

This paraquat concentration is high enough to see clear differences in sensitivity between strains, but low enough that most of the strains are still able to grow. For each tested strain, one colony was inoculated in duplicate in YP 2% (w/v) glycerol + 1 µM CuSO_4_, supplemented with 0.125 mM paraquat and serially diluted for growth overnight at 30 °C and 900 rpm. Copper was added to improve respiratory growth (Schlecht et al. 2014). Cultures at OD_600_ < 0.1 were transferred to 150 µl of fresh medium and serially diluted for growth for two overnights. Finally, cultures at OD_600_ < 0.1 were selected and used to inoculate in technical duplicate for growth measurements in a Bioscreen C device (resulting in four growth measurements per strain). The two consecutive precultures ensure that every culture is fully adapted and growing exponentially before the start of the growth experiment. Cells were incubated at 30 °C, with continuous medium shaking and OD_600_ was tracked every 15 min. Growth rates were determined using an in-house python script, and final OD_600_ measurements were obtained after 120 h of growth.

### *Petite* Frequency Assays

*Petite* frequency assays were performed as described in Dimitrov et al. (2009). In short, for each tested strain, one colony was inoculated in 3 ml YP 2% (w/v) glucose in quintuplicate and grown for one overnight at 30 °C. Appropriate dilutions were plated onto YP 0.1% (w/v) glucose + 3% (w/v) glycerol plates and the number of small (*petite*) and big (*grande*) colonies was counted after growth for 5 days at 30 °C.

### Experimental Evolution

For each deletion strain, four replicate starting populations were established (eight for the wild type) by inoculating single colonies in different wells of a 96-well plate containing 100 µl YP 2% (w/v) glucose. Outer wells were not inoculated, just as well as six wells in between inoculated wells to get an early indication on cross-contamination. After one overnight, 1 µl from each well was transferred to new plates containing 100 µl of selective medium: YP 2% (w/v) glycerol supplemented with 0.125 mM paraquat and YP 2% (w/v) glucose supplemented with 1 mM paraquat. Medium containing glycerol as a carbon source was always supplemented with 1 µM CuSO_4_. Populations were then evolved by serial dilution, using a dilution factor of 1:100. Transfers were done every 2 days for strains evolving on YP 2% (w/v) glucose with paraquat, and every 4 days for strains evolving on YP 2% (w/v) glycerol with paraquat. OD_600_ was measured daily to keep track of general adaptation trends and possible contamination. Glycerol stocks were made every 8 days. For strains evolving on YP 2% (w/v) glucose with paraquat, the paraquat concentration was increased by 0.5 mM every four transfers, and for strains evolving on YP 2% (w/v) glycerol with paraquat, the paraquat concentration was increased by 0.125 mM every eight transfers. As these conditions were too harsh for some of the most sensitive strains, they were put on a separate plate with an adjusted paraquat concentration regiment (for YP 2% (w/v) glucose: starting paraquat concentration of 0.5 mM, increased every eight transfers with 0.5 mM; for YP 2% (w/v) glycerol: starting paraquat concentration of 0.0625 mM, increased every 16 transfers with 0.0625 mM). To test whether the difference in concentration regiment influences the outcome of evolution, the plates with hypersensitive strains also included an additional four replicates of the wild-type strain. After evolution, we determined the effect of the concentration regiment on the growth characteristics of a subset of the evolved strains by measuring their growth characteristics at different concentrations of paraquat. Neither the concentration regiment nor the original sensitivity of the strain had an effect on how the strains behave at different paraquat concentrations (supplementary fig. 6*A*, Supplementary Material online). In addition to the four replicates of the wild-type strain, our data set also contains a number of other strains for which a number of replicates were evolved in the regular concentration regime, whereas other replicates of the same genetic background were evolved at the lower concentration. When we compare the growth rates of the evolved strains in both regimes, we do not observe a significant difference (supplementary fig. 6*B*, Supplementary Material online). The experiment was stopped after 150 generations, as most of the populations by then showed a significant increase in fitness (as estimated by looking at the daily increase in OD_600_). The number of generations was determined by estimating population size for each well based on the OD_600_ measurements upon transfer to fresh medium.

### Sampling Fit Clone from Evolved Populations

One fit evolved yeast clone was isolated from each evolved population. Plates with evolved populations were thawed, and 5 µl was inoculated in 150 µl YP 2% (w/v) glucose for overnight growth. Three microliters of appropriate dilutions was spotted on plates containing the same growth medium as was used for evolution: Strains evolved on YP 2% (w/v) glycerol supplemented with paraquat were spotted on plates containing YP 2% (w/v) glycerol supplemented with 0.125 mM paraquat and strains evolved on YP 2% (w/v) glucose supplemented with paraquat were spotted on plates containing YP 2% (w/v) glucose supplemented with 1 mM paraquat. Plates were incubated at 30 °C until single colonies could be clearly distinguished. From each spot, three big colonies were selected and inoculated in 150 µl fresh YP 2% (w/v) glucose medium for overnight growth. Using a Singer Rotor HDA pinning robot, all isolates were spotted on agar plates containing the same growth medium as used for evolution (as described above). Plates were incubated at 30 °C and were scanned daily using a high-definition scanner (Epson). Images were analyzed using CellProfiler version 2.2.0 (Lamprecht et al. 2007), and based on the area of the spots over time growth rates were calculated. The clone with the highest growth rate out of the three selected clones was selected for sequencing and further phenotyping. To verify that with this procedure we pick a clone that is a fair representative for the population, we compared the growth characteristics of the evolved bulk populations with those of the corresponding evolved clones (in liquid culture, for a subset of 16 population-clone pairs). There is a clear correlation between the growth rate of the clones and the growth rate of the populations from which they were picked (*r* = 0.86, *P* < 0.0001), indicating that the picked clones are not just rare outliers within the population (supplementary fig. 7, Supplementary Material online).

### Phenotypic Screens

Growth of each of the selected clones was measured under a variety of stresses, which were selected to influence a broad range of cellular processes. A complete list of compounds that were used and their concentrations can be found in supplementary table 11, Supplementary Material online. Plates with selected clones were thawed, and 5 µl of each strain was inoculated in 100 µl YP 2% (w/v) glucose for overnight growth. Using a Singer Rotor HDA pinning robot, all isolates were spotted on agar plates containing the drug or condition of choice. Plates were incubated at 30 °C and were scanned daily using a high-definition scanner (Epson). Images were analyzed using CellProfiler version 2.2.0 (Lamprecht et al. 2007), and based on the area of the spots over time growth rates were calculated. Growth rates were normalized for growth on the control condition (YP 2% (w/v) glucose), except for conditions where another carbon source was used. The growth rate of the evolved clones was then compared with the growth rate of their corresponding ancestor (same gene deletion) to calculate their increase or decrease in fitness.

### Determining Cell Ploidy

Cell ploidy of the selected evolved clones was determined by staining the cells with propidium iodide. For each sample, fluorescence of 50,000 cells was analyzed by flow cytometry on an Attune NxT. A prototrophic haploid strain (FY4) and an isogenic diploid strain (FY4/FY5) were used for calibration.

### Whole-Genome Sequencing and Variant Calling

#### Sample Preparation and Sequencing

Using a standard zymolyase-based protocol, genomic DNA was isolated from the ancestral wild type (FY4 HAP1+ SAL1+ MIP1+) and each of the selected evolved clones. Final DNA concentrations were measured with a Qubit 2.0 and DNA quality was checked using a NanoDrop 8000 and by gel electrophoresis. Samples were sent for paired-end sequencing on an Illumina HiSeq, with an average read length of 150 bp and an average insert size of 350 bp. Each of the samples had a minimum haploid coverage of 100×.

#### Mapping and Variant Calling

The general quality of the reads was assessed using FastQC version 0.11.5 (Babraham Bioinformatics) after which reads were mapped to the reference S288c genome (version R64) using bwa-mem version 0.7.12 with default settings (Li and Durbin 2010). Indels and SNVs were called using GATK version 4.0.0.0 (McKenna et al. 2010), according to GATK best practice recommendations. Duplicates were marked and HaplotypeCaller was used to call variants while setting ploidy to the level determined by propidium iodide staining. Regions with aneuploidies were later recalled using the adjusted local ploidy. Variants present in the ancestral strain were filtered out, as well as SNVs that were identical in more than three independent samples, and indels identical in more than one independent sample. All of the variants that were filtered out in this way were ambiguous calls, as determined by manual curation. Finally, all remaining SNVs and indels were verified using intensive manual curation.

#### Identification of Copy Number Variants

Structural variants were called using CNVnator version 0.3.3 (Abyzov et al. 2011), using a bin size of 250 bp. The output was verified using chromosomal density plots.

### Statistical Analyses

1. To test the hypothesis that all strains have at most the expected growth rate of the wild type, we used a simulation approach. First, as a statistic the sum of *z*-scores over every data point was used. To calculate the *z*-score, we took the wild type’s average growth rate and standard deviation (SD). As we were only testing if the fitness is higher than expected for some strains, only positive *z*-scores were considered and negative *z*-scores were set to zero. Growth rate residuals were normally distributed in our observations. The H0 distribution was approximated by calculating the statistic for simulated data sets that match the composition of our observations (10k repetitions) by sampling observations from normal distributions for every strain with the wild-type expected growth rate and SD. Next, this distribution was compared with the statistic calculated based on our observations. To be confident on the robustness of the observed test statistic, that is, does the result not depend on a handful of outliers, we estimated the variability in the statistic by calculating a bootstrap distribution for our observations.
2. *Modeling growth rate after evolution*. The number of genetic and physical interactions of each gene, as well as the number of phenotypes, and the information on whether the gene has a paralog and is part of a complex were obtained from Saccharomyces Genome Database using the Yeastmine tool. The conservation score was calculated as the average PhastCon score over the length of the gene. The PhastCon score for each nucleotide was retrieved from UCSC using the sacCer3 genome assembly. To estimate the effect of each biological parameter on growth rate after evolution, a linear model was fitted in R with different biological parameters as independent variables. The explained variance and significance of every variable were estimated by ANOVA, comparing the full model to the model without the variable. The plot of the model was made using the R package itools, and it represents the standardized coefficients with their 95% confidence intervals.
3. *Mann–Whitney U tests*. The Mann–Whitney *U* tests were performed using the “manwhitneyu” function from the stats module of SciPy (Virtanen et al. 2020).
4. *GO enrichment of mutations*. The overall GO enrichment of mutations across all samples was done using the competitive gene set test “camera,” from the R package edgeR (Wu and Smyth 2012). For the GO categories that were obtained in this overall test, targeted tests using the camera function were performed for each network module against the background.

### Phenotypic and Mutational Distances

The phenotypic profile of each evolved strain was defined as the relative increase in fitness compared with its corresponding unevolved deletion strain across each tested condition (see phenotypic screens above for more details). To calculate phenotypic distances, the average Euclidean distance was calculated between each strain and all other strains that came from the same deletion ancestor.

The mutational profile of each evolved strain was defined as its set of genes and GO categories that were mutated among the list of all the genes and GO categories that were mutated at least once across all the samples. To calculate mutational distances, the average binary (Jaccard) distance was calculated between each strain and all other strains that came from the same deletion ancestor.

Values for the phenotypic potential were taken from Levy and Siegal (2008). Two outliers were identified and removed using the ROUT method (*Q* = 1%).

## Data Availability

Aligned sequences of evolved clones have been deposited at ENA under accession number PRJEB39189.

## Supplementary Material

msaa172_supplementary_data
