# Supplementary material for: Gene Loss Predictably Drives Evolutionary Adaptation
Source: Mol Biol Evol. 2020 Jul 13;37(10):2989–3002. doi: 10.1093/molbev/msaa172 (PMC7530610; doi:10.1093/molbev/msaa172)
Supplement: msaa172_supplementary_data [file msaa172_supplementary_data.zip › Supplementary_Figures.pdf]

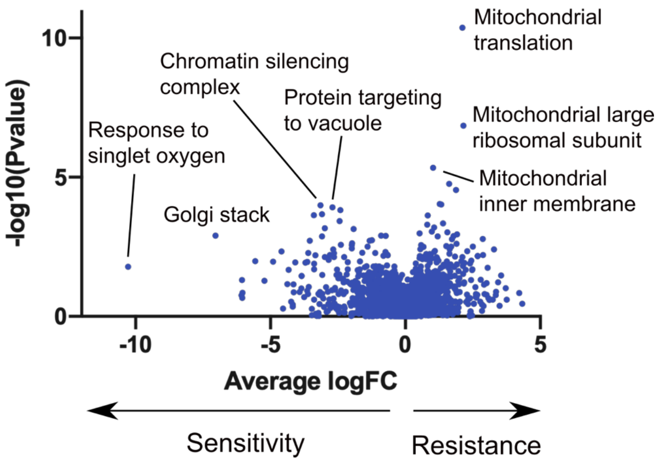

Supplementary Fig. 1 | GO enrichment of the most depleted and enriched processes under paraquat stress. GO analysis showing the processes with the strongest depletion and enrichment under paraquat stress. The x-axis represents the average log fold change of all deletion strains within a particular GO category. Deletions of genes within GO categories with a negative logFC confer sensitivity to paraquat, whereas deletions of genes belonging to GO categories with a positive logFC increase resistance. The y-axis represents the p-value of a competitive gene set overrepresentation test as calculated by the camera function of the edgeR package.

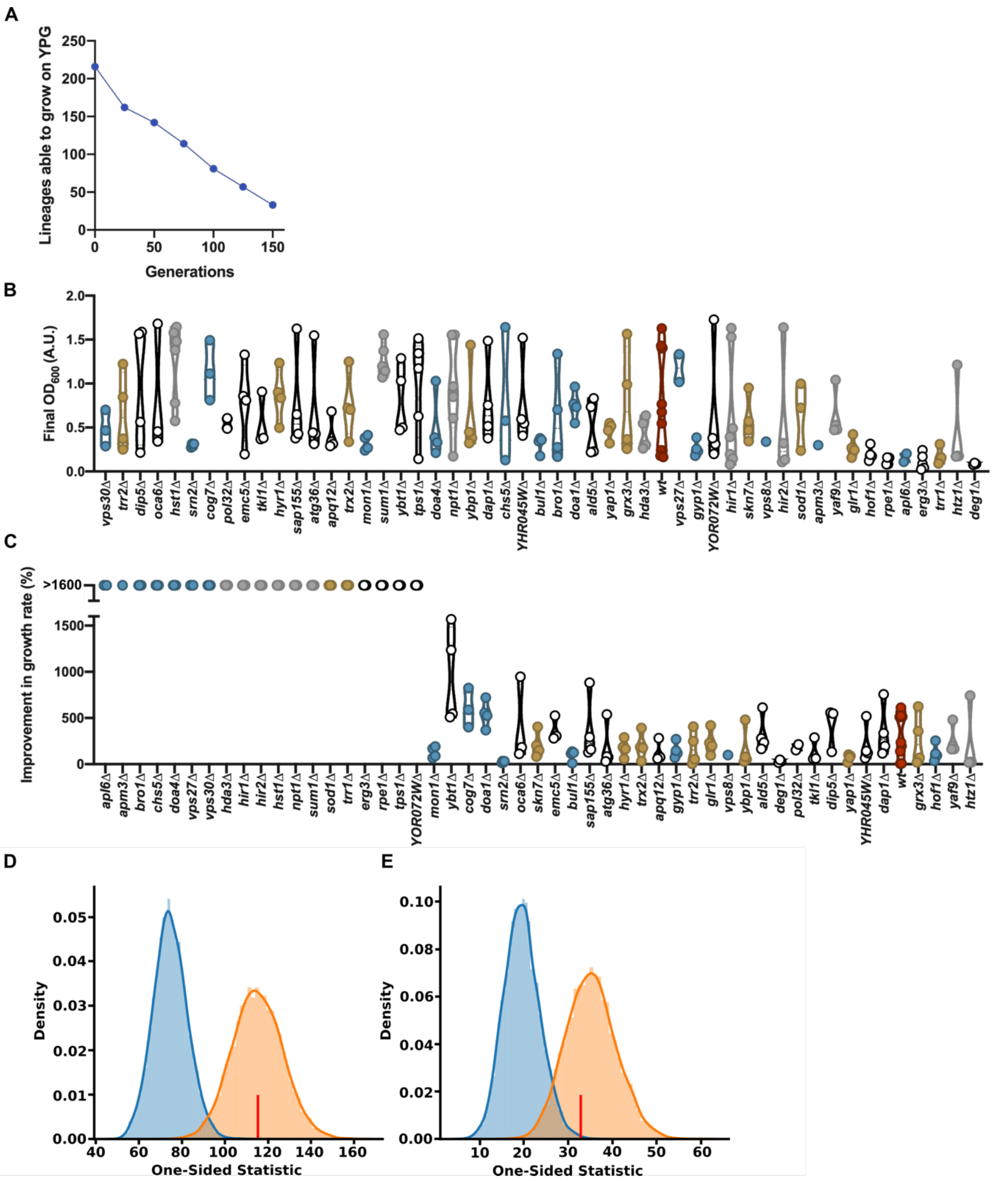

Supplementary Fig. 2 | Loss of mitochondrial function, final OD_{600} after evolution and testing whether deletion strains acquire higher growth rates than the wild type. (A) The number of independent lineages evolving on rich medium with 2% (w/v) glucose and paraquat that are still able to grow on rich medium with 2% (w/v) glycerol in function of the number of generations. Lineages that fail to grow on this medium presumably lost their mitochondrial

function. **(B)** Final OD_{600} after 120h of growth of one representative clone for each evolved population on rich medium with 2% (w/v) glycerol and 0.125 mM paraquat. Each point represents the mean of four replicate measurements. **(C)** Improvement in the final OD_{600} reached after 120h growth on rich medium with 2% (w/v) glycerol and 0.125 mM paraquat of one representative clone for each evolved population. **(D)** Histogram with fitted density distributions for simulation test for higher than wild-type average fitness. The distribution for the simulated null hypotheses is shown in blue. The bootstrap distribution of the test statistic based on the observed data is shown in orange. The calculated statistic based on the observed data is given as a red bar. Analysis on the full dataset shows that the null hypothesis can be robustly rejected and does not depend on a few outliers ($p < 0.005$ threshold outside the estimated 95% confidence interval for the test statistic). The average fitness after evolution for some deletion strains is higher than the average fitness of the wild-type. **(E)** Analysis for a subset of the data representing deletion strains with initial fitness similar to the wild-type. We selected *apq12*Δ, *hyr1*Δ, *yap1*Δ, *hof1*Δ, YHR045WΔ, *trr2*Δ, *dap1*Δ, *htz1*Δ, *grx3*Δ, *tkl1*Δ, *pol32*Δ, *yaf9*Δ and *dip5*Δ. Again the null hypothesis is rejected ($p < 0.005$ threshold outside the estimated 95% confidence interval for the test statistic). The evolution regime and low initial fitness of the deletion strain do not influence the conclusion that deletion strains can achieve higher than wild-type average fitness.

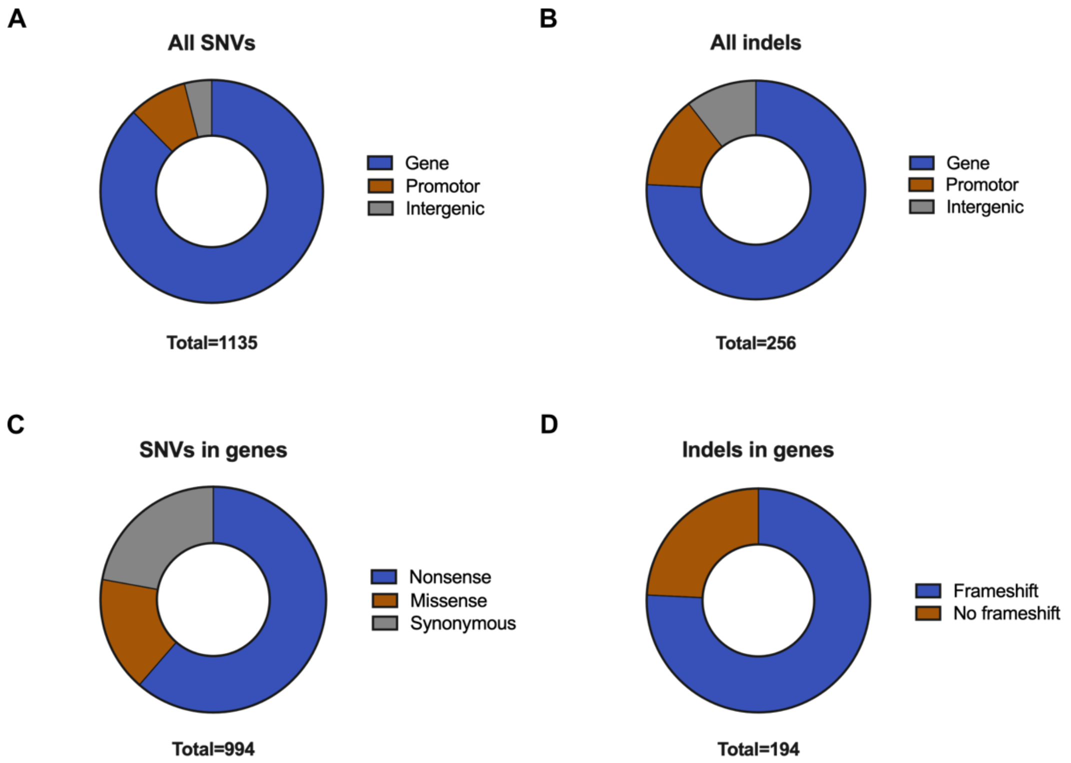

Supplementary Fig. 3 | Summary of types of mutations after evolution. (A) Proportion of SNVs that fall into genes, promoters and intergenic regions. **(B)** Proportion of indels that fall into genes, promoters and intergenic regions. **(C)** Effect of SNVs within genes. **(D)** Effect of indels within genes.

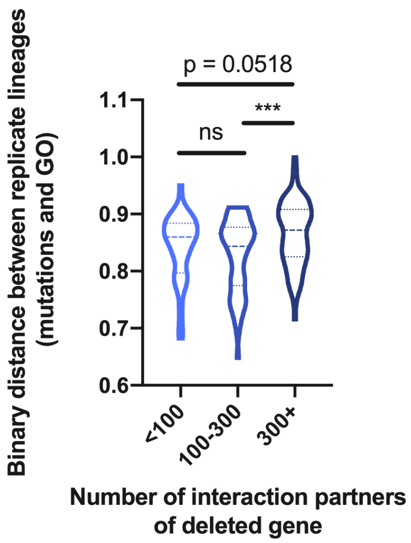

Supplementary Fig. 4 | Mutational distance between replicate lineages in function of the number of genetic interactions of the deleted gene. The mutational profile is defined by the genes and GO categories that were mutated after evolution. A high value for the mutational distance corresponds with more variation between the mutational profiles of the replicate lineages of a particular deletion strain. *** $p < 0.001$ (one-way ANOVA with Tukey's multiple comparison test)

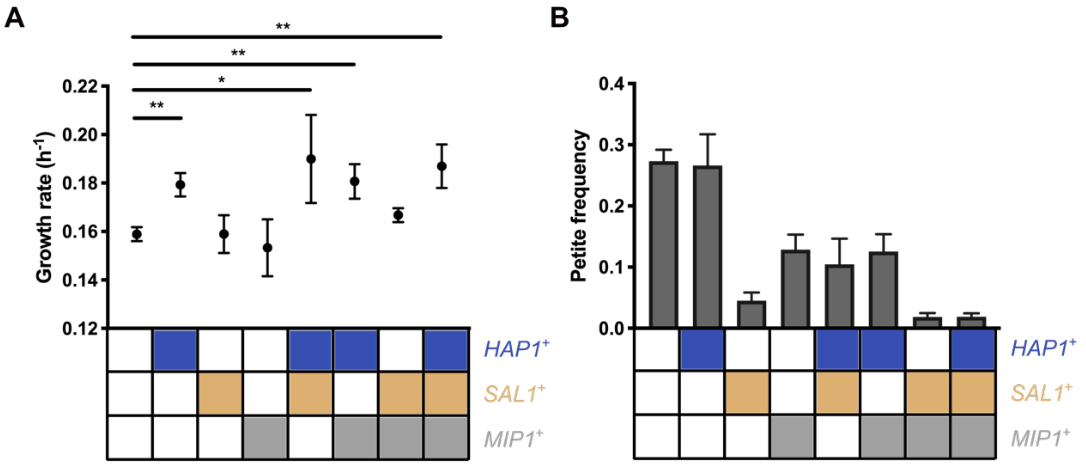

Supplementary Fig. 5 | Improving mitochondrial function and mitochondrial genome stability by repairing *HAP1, SAL1* and *MIP1*. (A) Growth rate of FY4 with varying combinations of repaired genes (*HAP1* (blue), *SAL1* (orange) and *MIP1* (grey)) on YP 2% (w/v) glycerol supplemented with 1 µM $CuSO_4$. Error bars represent standard deviations of three biological replicates. * $p < 0.05$, ** $p < 0.01$ (Student's t test). **(B)** *Petite* frequency of FY4 with varying combinations of repaired genes (*HAP1* (blue), *SAL1* (orange) and *MIP1* (grey)). Error bars represent standard deviations of five biological replicates.

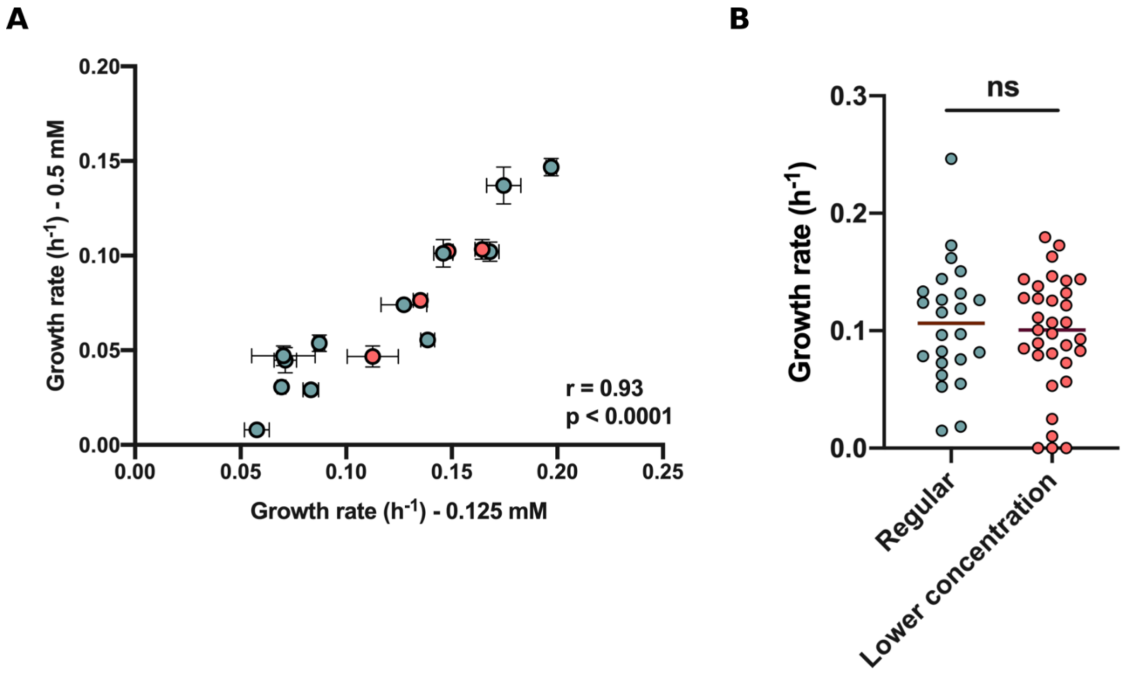

Supplementary Fig. 6 | The effect of the concentration regiment on growth rate after evolution. (A) Growth rates of 16 evolved clones at low and high paraquat concentration. Blue dots represent clones that come from the ‘regular evolution regime, Red dots represent clones that were evolved at a lower concentration. Error bars represent the standard deviation of four replicate measurements. The strong correlation ($r=0.93$, $p < 0.0001$) between growth rates at different paraquat concentrations indicates that we can fairly compare between strains. **(B)** Growth rates of evolved clones (YPG + 0.125 mM paraquat) for which some replicates coming from the same genetic background were evolved following the ‘regular’ concentration regime and others at the lower concentration regime. There is no significant difference.

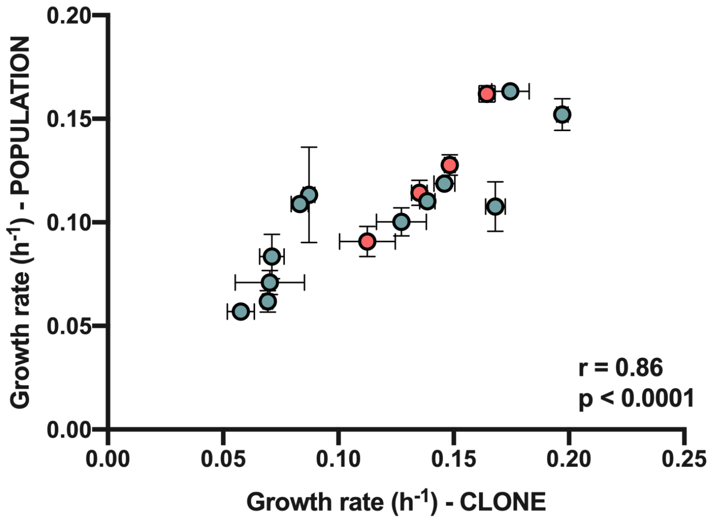

Supplementary Fig. 7 | Growth rates of 16 evolved clones and their corresponding populations under paraquat stress. Blue dots represent clones that come from the ‘regular evolution regime, Red dots represent clones that were evolved at a lower concentration. Error bars represent the standard deviation of four replicate measurements. Growth measurements were done in liquid culture (YPG + 0.125 mM paraquat).
